# Indoleamine 2,3-Dioxygenase Cannot Inhibit *Chlamydia trachomatis* Growth in HL-60 Human Neutrophil Granulocytes

**DOI:** 10.3389/fimmu.2021.717311

**Published:** 2021-11-08

**Authors:** Dezső P. Virok, Ferenc Tömösi, Anikó Keller-Pintér, Kitti Szabó, Anita Bogdanov, Szilárd Poliska, Zsolt Rázga, Bella Bruszel, Zsuzsanna Cseh, Dávid Kókai, Dóra Paróczai, Valéria Endrész, Tamás Janáky, Katalin Burián

**Affiliations:** ^1^ Department of Medical Microbiology, Albert Szent-Györgyi Health Center and Faculty of Medicine, University of Szeged, Szeged, Hungary; ^2^ Department of Medical Chemistry, Interdisciplinary Centre of Excellence, University of Szeged, Szeged, Hungary; ^3^ Department of Biochemistry, University of Szeged, Szeged, Hungary; ^4^ Department of Biochemistry and Molecular Biology, University of Debrecen, Debrecen, Hungary; ^5^ Department of Pathology, University of Szeged, Szeged, Hungary

**Keywords:** *Chlamydia*, *Chlamydia trachomatis*, IDO, interferon, neutrophil, polymorphonuclear (PMN), granulocyte

## Abstract

**Aims:**

Neutrophil granulocytes are the major cells involved in *Chlamydia trachomatis* (*C. trachomatis*)-mediated inflammation and histopathology. A key protein in human intracellular antichlamydial defense is the tryptophan-degrading enzyme indoleamine 2,3-dioxygenase (IDO) which limits the growth of the tryptophan auxotroph *Chlamydia*. Despite its importance, the role of IDO in the intracellular defense against *Chlamydia* in neutrophils is not well characterized.

**Methods:**

Global gene expression screen was used to evaluate the effect of *C. trachomatis* serovar D infection on the transcriptome of human neutrophil granulocytes. Tryptophan metabolite concentrations in the *Chlamydia*-infected and/or interferon-gamma (IFNG)-treated neutrophils were measured by ultra-high-performance liquid chromatography–tandem mass spectrometry (UHPLC–MS/MS).

**Results:**

Our results indicate that the *C. trachomatis* infection had a major impact on neutrophil gene expression, inducing 1,295 genes and repressing 1,510 genes. A bioinformatics analysis revealed that important factors involved in the induction of neutrophil gene expression were the interferon-related transcription factors such as IRF1-5, IRF7-9, STAT2, ICSB, and ISGF3. One of the upregulated genes was *ido1*, a known infection- and interferon-induced host gene. The tryptophan-degrading activity of IDO1 was not induced significantly by *Chlamydia* infection alone, but the addition of IFNG greatly increased its activity. Despite the significant IDO activity in IFNG-treated cells, *C. trachomatis* growth was not affected by IFNG. This result was in contrast to what we observed in HeLa human cervical epithelial cells, where the IFNG-mediated inhibition of *C. trachomatis* growth was significant and the IFNG-induced IDO activity correlated with growth inhibition.

**Conclusions:**

IDO activity was not able to inhibit chlamydial growth in human neutrophils. Whether the IDO activity was not high enough for inhibition or other chlamydial growth-promoting host mechanisms were induced in the infected and interferon-treated neutrophils needs to be further investigated.

## Introduction

*Chlamydia trachomatis* is an obligate intracellular bacterium that causes a variety of medically important diseases, including conjunctivitis, trachoma, pelvic inflammation, infertility, and lymphogranuloma venereum (1). The histopathological background of these diseases includes a profound acute inflammation that frequently leads to chronic inflammation and fibrosis. It is well known that neutrophil granulocytes or polymorphonuclear leukocytes play an important role in the *Chlamydia*-mediated acute inflammatory process. In animal models, the most abundant leukocyte cell type was the neutrophil granulocyte during early *Chlamydia* infection (2). The presence of neutrophils was also observed in *C. trachomatis*-infected human endocervical samples (3). Conjunctival samples taken from children with active trachoma also showed the expression of various genes that could be linked to neutrophils (4). While there are conflicting results concerning the role of neutrophils in suppressing *Chlamydia* growth (5), neutrophils are considered as being a major source of *Chlamydia* infection-induced tissue damage and remodeling (6).

A key factor in human intracellular defense against *Chlamydia* infection is the infection- and IFNG-induced host indoleamine 2,3-dioxygenase (IDO) activity (7, 8). IDO is a rate-limiting enzyme in the kynurenine pathway of tryptophan catabolism. Restricting the availability of tryptophan for *Chlamydia* is, in theory, an effective defense mechanism that can work in human cells, and it may work in murine cells also (9). Besides pattern recognition by the host cells, IFNG produced by T cells (10) and NK cells (11) is a key cytokine that is involved in the upregulation of IDO expression in *Chlamydia-*infected cells (8). While the defensive role of IDO was described mostly in *Chlamydia*-infected epithelial cells (7), according to the Human Protein Atlas, IDO is produced by a variety of other cell types, such as endothelial cells, fibroblasts, lymphocytes, monocytes/macrophages, dendritic cells, and neutrophils (12). The induction and potential role of IDO in neutrophil antichlamydial intracellular defense is less described. Here we investigated the global gene expression altered by *C. trachomatis* infection in the human neutrophil cell line HL-60, and we detected extensive host gene expression changes. The *ido1* gene was found among the upregulated genes with 8.31-fold of upregulation. We characterized the activity of IDO in *Chlamydia-*infected and IFNG-treated HL-60 cells and as a control in HeLa human cervical epithelial cells. We detected major differences in the IFNG-induced IDO activity and IFNG-induced chlamydial growth suppression in HL-60 cells and HeLa cells.

## Materials and Methods

### Chlamydia Strain

*C. trachomatis* serovar D strain UW-3/CX was propagated in HeLa 229 cells. Infectious chlamydial elementary bodies were purified by density gradient centrifugation, and inclusion-forming units (IFU) were determined as described previously (13). A mock sample was prepared from uninfected HeLa cell monolayer processed in the same way as the infected cells.

### Cell Culture and Infection

HL-60 cells were maintained in RPMI-1640 medium supplemented with 10% v/v heat-inactivated fetal bovine serum (FBS; Gibco, Germany), 2 mmol/l of L-glutamine, 8 mmol/l HEPES, 25 μg/ml gentamycin, and 1 µg/ml fungizone under humidified air containing 5% CO_2_ at 37°C. The HL-60 cells were differentiated in culture medium supplemented with 12.5 μl/ml dimethyl sulfoxide for 5 days (14). The differentiated HL-60 cells were infected with *C. trachomatis* serovar D at a multiplicity of infection (MOI) of 4 or an identical volume of mock sample for 1 h in 0.5% glucose containing medium without centrifugation. After infection, the HL-60 cells were washed twice with phosphate-buffered saline (PBS), and culture medium without cycloheximide was added. For microarray studies, tryptophan catabolism measurements, and Western blot, HL-60 cells infected in six-well plates (1 × 10^6^ cells in 3 ml medium) were washed twice with PBS and collected at 24 h post-infection (parallel measurements were performed; *n* = 3 for microarray analysis, *n* = 4 for tryptophan catabolism measurements, and *n* = 3–4 for Western blot). For cell viability assays and direct and recoverable chlamydial growth measurements, HL-60 cells were infected in 96-well plates (4 × 10^4^ cells in 0.1 ml medium), washed twice with PBS, and collected at 24 h (cell viability assays) or 48 h post-infection in sucrose–phosphate–glutamic acid buffer (SPG) or analyzed (parallel measurements were performed; *n* = 8 for 3-(4,5-Dimethylthiazol-2-yl)-2,5-diphenyltetrazolium bromide (MTT) assays, *n* = 4 for viable cell counting, and *n* = 5 for chlamydial growth measurements).

HeLa 229 cells were maintained in minimal essential medium (MEM) with Earle salts supplemented with 10% heat-inactivated FBS (Gibco), 2 mmol/l L-glutamine, 1× MEM vitamins, 1× non-essential amino acids, 25 μg/ml gentamycin, and 1 µg/ml fungizone. The HeLa cells were infected with *C. trachomatis* (MOI, 4) for 1 h in 0.5% glucose containing medium without centrifugation. After infection, the HeLa cells were washed twice with PBS, and culture medium without cycloheximide was added. For the microarray studies, tryptophan catabolism analysis, and Western blot, HeLa cells infected in six-well plates (1 × 10^6^ cells in 3 ml medium) were washed twice with PBS and collected at 24 h post-infection (parallel measurements were performed; *n* = 3 for microarray analysis, *n* = 4 for tryptophan catabolism analysis, and *n* = 3–4 for Western blot). For cell viability assays and direct and recoverable chlamydial growth measurements, HeLa cells infected in 96-well plates (4 × 10^4^ cells in 0.1 ml medium) were washed twice with PBS and collected at 24 h (cell viability assays) or 48 h post-infection in SPG or analyzed (parallel measurements were performed; *n* = 8 for MTT assays, *n* = 4 for viable cell counting, and *n* = 5 for chlamydial growth measurements).

For IFNG-induced IDO activity measurements and chlamydial growth suppression experiments, recombinant human IFNG (PeproTech, London, UK) was diluted in culture medium without cycloheximide. IFNG was added to the cells immediately after the infection. For tryptophan degradation measurements, Western blot assay, and transmission electron microscopy (TEM), 20 IU/ml IFNG was used. For cell viability and chlamydial growth monitoring experiments, 20, 40, and 80 IU/ml IFNG were added.

### Determination of Recoverable *C. trachomatis* Growth on McCoy Cells

McCoy cells were transferred into the wells of the 96-well plate with a density of 4 × 10^4^ cells/well in 100 µl of MEM and were incubated overnight at 37°C and 5% CO_2_ to get a 90% confluent cell layer. Before the infection, the wells were washed twice with 100 μl/well of PBS. After washing, 90 µl of the culture medium with 0.5% glucose was added to each well. For the determination of the recoverable *C. trachomatis* growth, 10 µl of the *C. trachomatis*-infected and the *C. trachomatis*-infected + IFNG-treated HeLa and HL-60 cell lysates produced by two freeze–thaw cycles in SPG were transferred onto the McCoy cells. The cells were centrifuged for 1 h at 800 × *g* and were incubated for 48 h in cycloheximide-containing (1 µg/ml) growth medium. All the cell culture reagents were purchased from Sigma (St. Louis, MO, USA) unless otherwise indicated.

### Microarray Hybridization and Data Analysis

Total RNA was extracted from *C. trachomatis*-infected and uninfected control HL-60 cells (*n* = 3) with Tri Reagent according to the instructions of the manufacturer (Sigma). Total RNA quantity (OD260) and quality (OD260/280) were measured by a NanoDrop Lite spectrophotometer (Thermo Scientific, Waltham, MA, USA). Affymetrix (Santa Clara, CA, USA) GeneChip Human PrimeView arrays were used to analyze global expression. The amplification and labeling of RNA was performed according to the protocol of the manufacturer. Briefly, 3’IVT Expression Kit (Affymetrix) and GeneChip WT Terminal Labeling and Control Kit (Affymetrix) were used for amplifying and labeling 250 ng of total RNA samples. The labeled cRNA samples were hybridized at 45°C for 16 h, then a standard washing protocol was performed using GeneChip Fluidics Station 450, and the arrays were scanned on GeneChip Scanner 7G (Affymetrix) and CEL files were generated. The CEL files were processed using Expression Console (Affymetrix) software to generate CHP files using Robust Multichip Average normalization algorithm. The CHP files were imported into Transcriptome Analysis Console 2.0 (Affymetrix) software to identify differentially expressed genes between the two conditions. To determine the statistical significance, an unpaired one-way ANOVA test was used with Benjamini–Hochberg false discovery rate (FDR) for correcting the multiple testing. The statistical significance was considered at FDR *p*-value <0.05 and a fold-change value ≥2.0. Gene Ontology biological function-based grouping and Kyoto Encyclopedia of Genes and Genomes (KEGG) pathway analysis of the differentially expressed genes were performed by the DAVID (15) and g:Profiler (16) online tools. Identification of enriched transcription factor binding sites in the promoter region of the significantly upregulated genes was performed by the g:Profiler online tool.

### Measurement of IDO1 Protein Expression by Western Blotting

HL-60 and HeLa cells (10^6^ cells/sample) were lysed in RIPA buffer (20 mM Tris-HCl, pH 7.5, 150 mM NaCl, 1 mM Na_2_EDTA, 1 mM EGTA, 1% NP-40, 1% sodium deoxycholate, 2.5 mM sodium pyrophosphate, 1 mM β-glycerophosphate, 1 mM Na_3_VO_4_, 1 μg/ml leupeptin; #9806, Cell Signaling Technology, Danvers, MA, USA) supplemented with protease inhibitor cocktail (Sigma-Aldrich, St. Louis, MO, USA), and samples were centrifuged at 13,000 rpm for 5 min at 4°C. Then, the protein concentrations of the supernatants were measured using a BCA assay kit (Pierce Chemical, Rockford, IL, USA). Equal amounts of proteins were separated on polyacrylamide gel and transferred onto Protran nitrocellulose membranes (GE Healthcare, Amersham, UK). After blocking with 5% non-fat dry milk, the membranes were incubated overnight with rabbit polyclonal anti-IDO1 (A1614; ABclonal, Woburn, MA, USA) and mouse monoclonal anti-alpha-tubulin (T9026; Sigma-Aldrich, St. Louis, MO, USA) antibodies. Then, the membranes were incubated with HRP-conjugated anti-rabbit (P0448) and anti-mouse (P0161) secondary antibodies (DAKO, Glostrup, Denmark). The peroxidase activity was detected using the enhanced chemiluminescence procedure (Advansta, Menlo Park, CA, USA). Signal intensities were quantified using the QuantityOne software program (Bio‐Rad, Hercules, CA, USA).

### UHPLC–MS/MS Reagents and Chemicals

See **Supplementary File 1**.

### Preparation of Standard, Internal Standard, and Quality Control Solutions

See **Supplementary File 1**.

### UHPLC–MS/MS Method Validation, Linearity, Limit of Detection, and Limit of Quantification

See **Supplementary File 1** and **Supplementary Tables 1**, **2**.

### UHPLC–MS/MS Methods for Targeted Metabolomics

The applied bioanalytical method optimization and its applicability for human body fluids (cerebrospinal fluid, serum, and plasma) were described in our previous publications (17, 18). The UHPLC separation of TRP and its metabolites was performed on an ACQUITY I-Class UPLC™ liquid chromatography system (Waters, Manchester, UK) consisting of Binary Solvent Manager, Sample Manager-FL, and Column Manager. The UPLC system was controlled using MassLynx 4.1 SCN 901 (Waters). Chromatographic separation for quantitative analysis of tryptophan and its 11 metabolites in the cell homogenate supernatant was performed at 25°C on a pentafluorophenyl (PFP) column (Phenomenex, Torrance, CA, USA; 100 Å, 100 × 2.1 mm, particle size 2.6 μm) protected by a PFP guard column (Phenomenex) using 0.1% (v/v) aqueous FA as solvent A and MeOH containing 0.1% (v/v) FA as solvent B. Then, 10 μl of the sample was injected into the UHPLC–MS/MS system. The mass spectrometric measurements were conducted using a Q Exactive™ Plus Hybrid Quadrupole-Orbitrap Mass Spectrometer (Thermo Fisher Scientific, San Jose, CA, USA) connected online to the UHPLC instrument as described previously (17). A divert valve placed after the analytical column was programmed to switch flow onto the mass spectrometer only when analytes of interest were eluted from the column (1.4–5.0 min) to prevent excessive contamination of the ion source and ion optics. The washing procedures of the autosampler before and after injecting the samples were programmed to avoid the carry-over of analytes.

### Preparation of HL-60 and HeLa Cell Homogenates for Targeted Metabolomics

Uninfected/untreated controls, *C. trachomatis*-infected, IFNG-treated, and infected+IFNG-treated cells were produced as described above. To remove cell culture media, HL-60 and HeLa cells were washed twice with PBS before processing. Prior to profiling the kynurenine and serotonin pathways, samples were relabeled, and hence a blind study was conducted. For the quantification of tryptophan and its metabolites, the HL-60 and HeLa cell lines were homogenized with an ultrasonic homogenizer in 120 μl PBS for 2 min on ice (with 2-s homogenization and 4-s resting cycles) and centrifuged after for 15 min at 15,000 × *g* at 4°C. Briefly, after centrifugation to 90 μl of each cell homogenate supernatant sample, 10 μl 0.1% (v/v) of aqueous FA and 300 μl of ice-cold ACN containing 10 μl of the SIL-IS mix (the same as that used in the preparation of the calibration standards) were added, and the mixture was vortexed for 30 s. Samples were incubated for 30 min at −20°C to support protein precipitation, and the supernatant was obtained *via* centrifugation of the mixture for 15 min at 15,000 × *g* at 4°C. The supernatant (390 μl) was transferred to a new tube, centrifuged for 15 s, and then split into two equal portions. After concentration in vacuum (Speed Vac Plus, Savant, RI, USA), half of the sample was treated with 70 μl of derivatizing reagent (n-butanol–acetyl chloride, 9:1, v/v) and incubated for 1 h at 60°C. The mixture was dried under nitrogen before reconstitution. Both parts of the sample were dissolved in 75 μl of the starting eluent, vortexed, centrifuged, and combined.

### Characterization of the Impact of IFNG on the Viability of HL-60 and HeLa Cells

MTT assay was performed to calculate the impact of IFNG on the viability of HL-60 and HeLa cells. Cell culture media were supplemented with 0, 20, 40, and 80 international unit/ml (IU/ml) IFNG, and the viability was assessed after 24 h of treatment as described earlier (19). The same experimental setup was used for viable cell count measurements. After 24 h of IFNG treatment, trypan blue exclusion method was used to count the viable cells. Cell counting was performed by a Countess 3FL automated cell counter (ThermoFisher, Waltham, MA, USA).

### Direct Quantitative PCR Measurement of *C. trachomatis* Genome Concentration

Measurement of chlamydial genome accumulation was used as a proxy to estimate *C. trachomatis* replication as described before (20). Briefly, the infected cells underwent two freeze–thaw cycles to make the chlamydial DNA accessible. The cell lysates were used directly as templates in the qPCR. Direct qPCR was applied to measure the relative chlamydial genome concentration in a Bio-Rad CFX96 real-time system. The qPCR mix contained the SsoFast™ EvaGreen^®^ qPCR Supermix (Bio-Rad, Hercules, CA, USA) master mix and the *C. trachomatis* primer pair *pykF*: 5’-GTTGCCAACGCCATTTACGATGGA-3’ and *pykR*: 5’-TGCATGTACAGGATGGGCTCCTAA-3’. For absolute quantitation, the *pyk* qPCR product was purified with GenElute PCR Clean-Up Kit (Sigma), and its concentration was determined by a NanoDrop Lite spectrophotometer (Thermo Scientific, Waltham, MA, USA). The *pyk* gene copy number in the purified qPCR product was calculated as described before (21). The chlamydial genome content of HL-60, HeLa, and McCoy samples was determined by a comparison of their *pyk* gene cycle threshold (Ct) levels to the Ct levels of samples which consisted of a known copy number of *pyk*-purified qPCR product diluted in HL-60, HeLa, and McCoy cell lysates as described before (21).

### TEM of IFNG-Treated and Infected HL-60 and HeLa Cells

HL-60 and HeLa cells were infected with *C. trachomatis* (MOI, 4) and were treated with 20 IU/ml IFNG or left untreated. At 48 h post-infection, the cells were pelleted and were fixed with 3% glutardialdehyde in PBS, pH 7.4. The specimens were embedded in Embed 812 (EMS, Hatfield, PA, USA), and 70-nm-thin sections were prepared with an Ultracut S ultra-microtome (Wetzlar, Germany). After staining with uranyl acetate and lead citrate, the sections were observed with a Jeol 1400 plus electron microscope (Freising, Germany).

### Statistical Analysis of UHPLC–MS/MS Data

The calculation of the peak area ratios and the calibration and quantitation of the analytes was performed from collected raw data using Xcalibur™ Quan Browser software (Thermo Fisher Scientific). The processed data for the peak area, peak area ratio, retention time, and concentrations were exported into Microsoft Excel software. The resulting concentrations of tryptophan and its metabolites were normalized to the cell numbers of the samples. An unpaired one-way ANOVA test was used, which was corrected for multiple comparisons by controlling the FDR by a two-stage linear step-up procedure of Benjamini, Krieger, and Yekutieli. The statistical significance was considered at FDR *P*-value <0.05.

### Statistical Analysis of *C. trachomatis* Growth and Western Blot Data

The *Chlamydia* genome copy numbers in untreated and IFNG-treated samples (*n* = 5) were compared by an unpaired one-way ANOVA test corrected for multiple comparisons by controlling the FDR by a two-stage linear step-up procedure of Benjamini, Krieger, and Yekutieli. The statistical significance was considered at FDR *P*-value <0.05. Quantified Western blot protein expression signal intensities were evaluated. Statistical differences between groups (*n* = 3-4) were analyzed by an unpaired one-way ANOVA test corrected for multiple comparisons by controlling the FDR by a two-stage linear step-up procedure of Benjamini, Krieger, and Yekutieli. The statistical significance was considered at FDR *P*-value <0.05. GraphPad Prism 9.2.0 software (GraphPad Software Inc., San Diego, CA, USA) was used for graphing and statistical analyses.

## Results

### Global Gene Expression Analysis of the *C. trachomatis*-Infected HL-60 Cells

**Figure 1:**
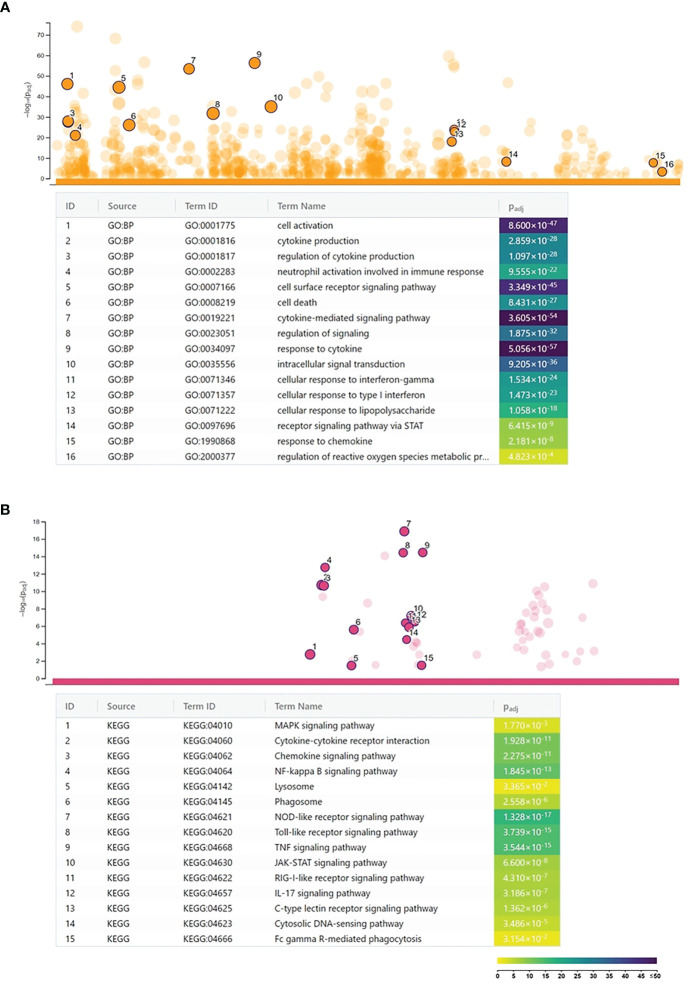
Functional analysis of the *Chlamydia trachomatis* infection-induced genes. **(A)** Gene Ontology analysis of significantly enriched biological function terms containing differentially expressed genes. The names and significance levels of enrichment of selected functional category terms are shown. **(B)** Analysis of significantly enriched Kyoto Encyclopedia of Genes and Genomes pathways containing induced genes. The names and significance levels of enrichment of selected pathways are shown. The color coding of biological function and pathway term enrichment *P*-values denotes the significance level of enrichment. The color scale shows the –log_10_ *P*-value of enrichment.

**Figure 2:**
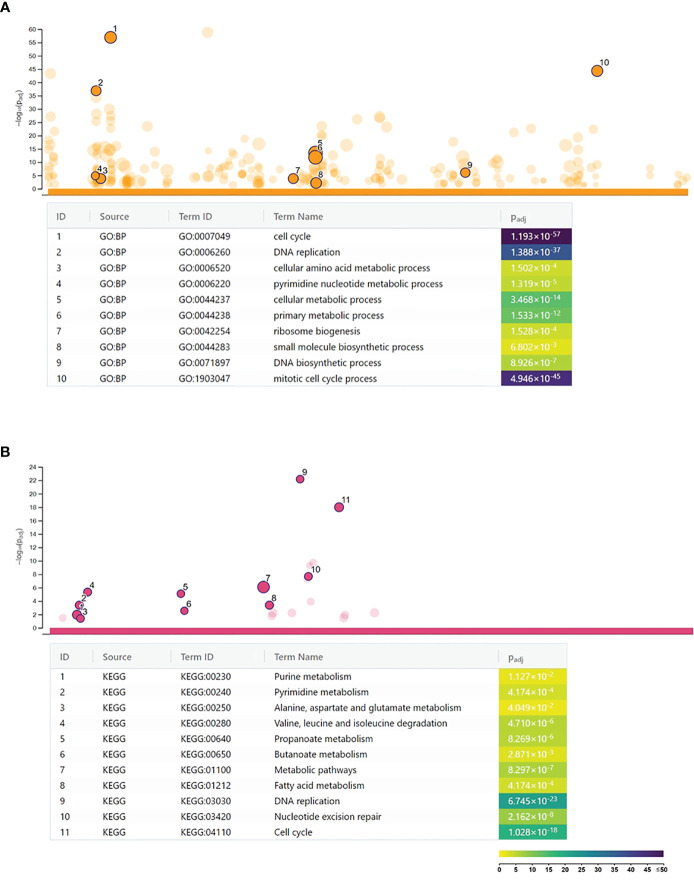
Functional analysis of the *Chlamydia trachomatis* infection repressed genes. **(A)** Gene Ontology analysis of significantly enriched biological function terms containing repressed genes. The names and significance levels of enrichment of selected functional category terms are shown. **(B)** Analysis of significantly enriched Kyoto Encyclopedia of Genes and Genomes pathways containing repressed genes. The names and significance levels of enrichment of selected pathways are shown. The color coding of biological function and pathway term enrichment *P*-values denotes the significance level of enrichment. The color scale shows the –log_10_ *P*-value of enrichment.

A microarray analysis was performed to get a global view on the impact of *C. trachomatis* infection on the gene expression changes of human neutrophils. Our results showed that *C. trachomatis* infection had a significant impact on neutrophil gene expression, inducing 1,295 genes and repressing 1,510 genes (≥twofold). The list of significantly upregulated and downregulated genes is presented in **Supplementary Table 3**. In order to get functional information on the altered genes, we performed a Gene Ontology biological function-based classification using the g:Profiler online analysis tool (16) (**Figure 1A**) (g:Profiler analysis data is included in **Supplementary Table 4**). Our analysis revealed that many of the enriched functional groups among the upregulated genes were related to neutrophil activation, such as “cellular response to lipopolysaccharide”, “regulation of reactive oxygen metabolic process”, and “neutrophil activation involved in immune response”. A second, even more prominent, theme was related to cytokine secretion and response to cytokines, indicating the infection-induced cytokine production and autocrine–paracrine response (signal transduction, gene expression induction) to these cytokines. Among these groups, “cytokine production”, “response to cytokine”, “response to interferon-gamma”, “response to type 1 interferon”, and “intracellular signal transduction” were found. KEGG pathway analysis by g:Profiler also found the “cytokine–cytokine receptor interaction”, “chemokine signaling pathway”, “TNF signaling pathway”, and “JAK-STAT signaling pathway” to be significantly enriched among the upregulated genes. Other pathways like “Toll-like receptor signaling pathway”, NOD-like and RIG-I-like receptor signaling pathways, and “NF-kappa B signaling pathway” were related to bacterial pattern recognition (**Figure 1B**). Enriched functional groups among the downregulated genes were mainly related to cell division and metabolism (**Figure 2A**). The KEGG pathway analysis also found various amino acid metabolism, lipid metabolism, nucleotide metabolism, and cell cycle-related pathways to be enriched among the downregulated genes (**Figure 2B**).

### Regulation of the Expression of Induced Genes in the *C. trachomatis*-Infected HL-60 Cells

**Figure 3:**
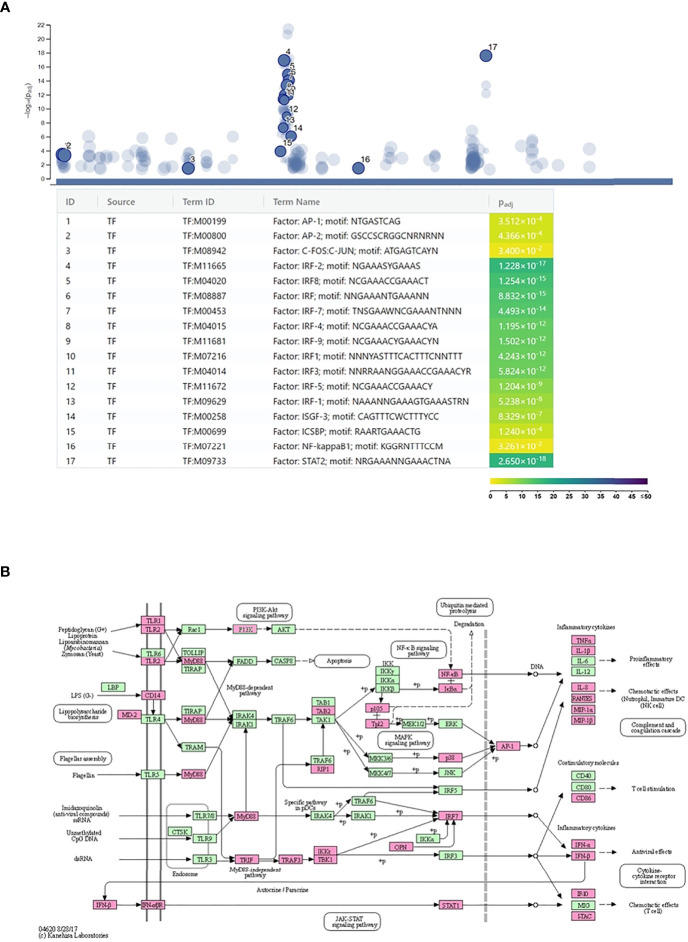
Regulation of *Chlamydia trachomatis* infection-induced neutrophil gene expression. **(A)** Identification of the enriched transcription factor binding sites among the promoters of the *C*. *trachomatis* infection-induced genes. The names and significance levels of enrichment of selected transcription factors are shown. The color coding of transcription factor binding site enrichment *P*-values represents the significance level of enrichment. The color scale denotes the –log_10_ *P*-value of enrichment. **(B)** *C. trachomatis* infection-induced genes mapped to the Kyoto Encyclopedia of Genes and Genomes Toll-like receptor signaling pathway. *C. trachomatis* infection-induced genes are shown in magenta.

The g:Profiler analysis of the promoter sequences of the upregulated genes identified several transcription factor-binding motifs to be highly significantly enriched in these sequences. We found inflammation-related transcription factors, such as AP1 (C-FOS, C-JUN) transcription factors and NF-kappaB, among the enriched ones. The most significantly enriched transcription factor motifs, such as IRF1-5, IRF7-9, STAT2, ICSB, and ISGF3, were related to IFN signaling (**Figure 3A**). Supporting these data, the transcription factor genes *stat1-3*, *stat6*, *irf1-2*, *irf7*, and *irf9* were found to be upregulated by *C. trachomatis* infection. These data indicate that self-produced IFNs had a major impact on the gene expression of neutrophils. The DAVID pathway analysis of the upregulated genes identified “Toll-like receptor signaling pathway” that contained a significant number of upregulated genes (Benjamini adjusted *P*-value: 3.9 × 10^-13^) and could lead to type-I IFN production (**Figure 3B**). Various members of this pathway, including type-I IFN genes themselves (*ifna1*, *ifna2*, *ifna8*, and *ifnb1*), were upregulated. These IFNs are able to bind to their receptors, such as the upregulated *ifnar1-2*, and induce the JAK-STAT cascade and eventually the expression of target genes. Among the target genes, we found the key antichlamydial gene *ido1* which is known to be induced by *Chlamydia* infection and interferon, especially IFNG (8).

### Impact of *C. trachomatis* Infection and IFNG on Tryptophan Degradation in HL-60 and HeLa Cells

**Figure 4:**
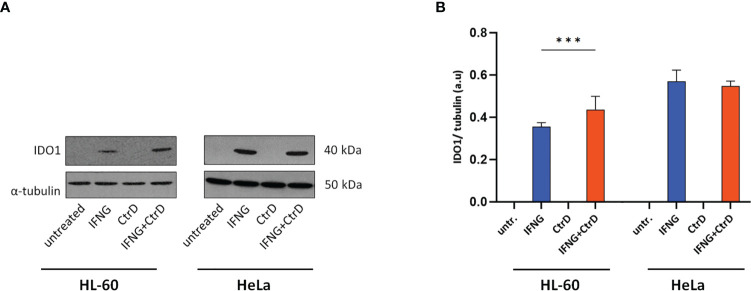
IDO1 expression in HL-60 and HeLa cells. Cells were infected with *Chlamydia trachomatis* (multiplicity of infection, 4) and were treated with 20 IU/ml interferon-gamma or left untreated. **(A)** Western blot was performed at 24 h post-infection. A representative Western blot is shown. **(B)** Western blot band intensities were quantified, and IDO1 expressions were normalized by α-tubulin expression. The normalized intensities in each cell type were compared by one-way ANOVA, with correction for multiple testing. Data are mean ± SD (*n* = 3–4). ***P < 0.001.

**Figure 5:**
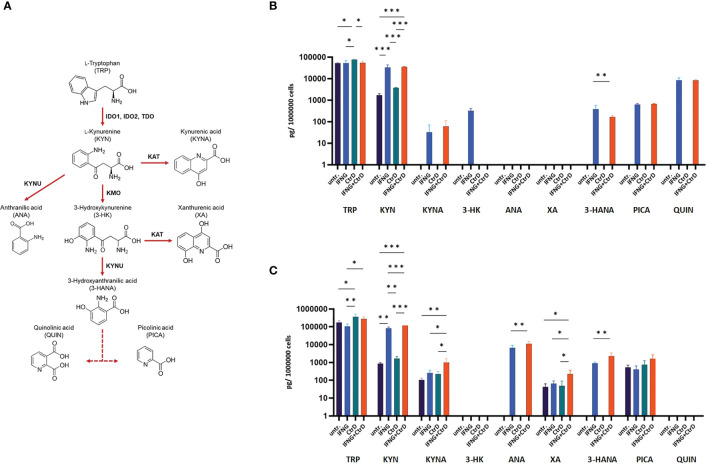
Impact of *Chlamydia trachomatis* infection and interferon-gamma (IFNG) treatment on the tryptophan catabolism of HL-60 and HeLa cells. **(A)** Simplified pathway of tryptophan catabolism, containing the principal enzymes kynurenine aminotransferase, kynurenine-3-monooxygenase, kynureninase, indoleamine-2,3-dioxygenase, and tryptophan-2,3-dioxygenase. **(B)** UHPLC–MS/MS measurement of tryptophan degradation products at 24 h post-infection/treatment in *C. trachomatis*-infected and/or IFNG-treated HL-60 cells. **(C)** UHPLC–MS/MS measurement of tryptophan degradation products at 24 h post-infection/treatment in *C. trachomatis*-infected and/or IFNG-treated HeLa cells. TRP, L-tryptophan; KYN, L-kynurenine; KYNA, kynurenic acid; 3-HK, 3-hydroxykynurenine; ANA, anthranilic acid; XA, xanthurenic acid; 3-HANA, 3-hydroxyanthranilic acid; PICA, picolinic acid; QUIN, quinolinic acid. Data are mean ± SD (*n* = 4). One-way ANOVA with correction for multiple testing was used to compare the metabolite concentrations between uninfected/untreated and infected and/or IFNG-treated cells. *P < 0.033; **P < 0.002; ***P < 0.001.

Western blot was used to determine whether *Chlamydia* infection and/or IFNG treatment induced IDO1 protein expression (**Figures 4A, B** **)**. As a control, HeLa cervical epithelial cells were used, which is permissive for *C. trachomatis*, and IFNG induces IDO expression in these cells (22). Uninfected/untreated and *C. trachomatis*-infected HL-60 and HeLa cells did not express IDO1. IFNG treatment induced IDO1 expression in both cell lines, indicating that IFNG is the major inducer of IDO1. Differently from HeLa cells and possibly due to infection-induced type-I IFN production, *C. trachomatis* infection significantly increased the IDO1 expression in IFNG-treated HL-60 cells compared to IFNG-only-treated cells. To test whether IDO1 was functional, we performed a UHPLC–MS/MS analysis of the uninfected/untreated and infected and/or IFNG treated HL-60 and HeLa cells at 24 h post-infection (**Figures 5A–C**). A low level of kynurenine production was detected in the uninfected/untreated HL-60 cells, which was not increased significantly by *C. trachomatis* infection. IFNG treatment and *C. trachomatis* infection+IFNG treatment greatly increased the tryptophan catabolism. The average kynurenine levels were 19.21-fold and 20.43-fold higher than in the uninfected/untreated cells, respectively. Despite the higher IDO1 protein expression in the infected+IFNG-treated cells than in the IFNG-treated cells, the kynurenine levels were not significantly different. Interestingly, *C. trachomatis* infection increased the total tryptophan level compared to the uninfected/untreated control cells. On the other hand, addition of IFNG to the *C. trachomatis*-infected cells significantly increased kynurenine production (9.56-fold compared to infected only) that led to a moderate but significant decrease in tryptophan concentration. Downstream metabolites, such as kynurenic acid, 3-hydroxyanthranilic acid, picolinic acid, and especially quinolinic acid, could be detected in the *C. trachomatis* infected+IFNG treated cells, but not in the infected-only cells. The HeLa cells showed a slightly different tryptophan degradation pattern. The uninfected/untreated cells produced a low level of kynurenine, but differently from HL-60, other downstream metabolites could also be detected at low concentrations. *C. trachomatis* infection induced an increase in tryptophan concentration. The addition of IFNG to the infected cells greatly increased the kynurenine concentration (131-fold), but it did not lead to a significant decrease in tryptophan concentration compared to the infected cells. Similarly to HL-60, the addition of IFNG to the infected cells resulted in a higher kynurenic acid and 3-hydroxyanthranilic acid production. Differently from HL-60 cells, the addition of IFNG to the infected cells induced anthranilic acid and xanthurenic acid production but could not induce a significant change in picolinic acid production. Anthranilic acid and xanthurenic acid production could not be detected in HL-60 cells, while 3-hydroxykynurenine and quinolinic acid production could not be detected in HeLa cells.

### Impact of IFNG on the Growth of *C. trachomatis* in HL-60 and HeLa Cells

**Figure 6:**
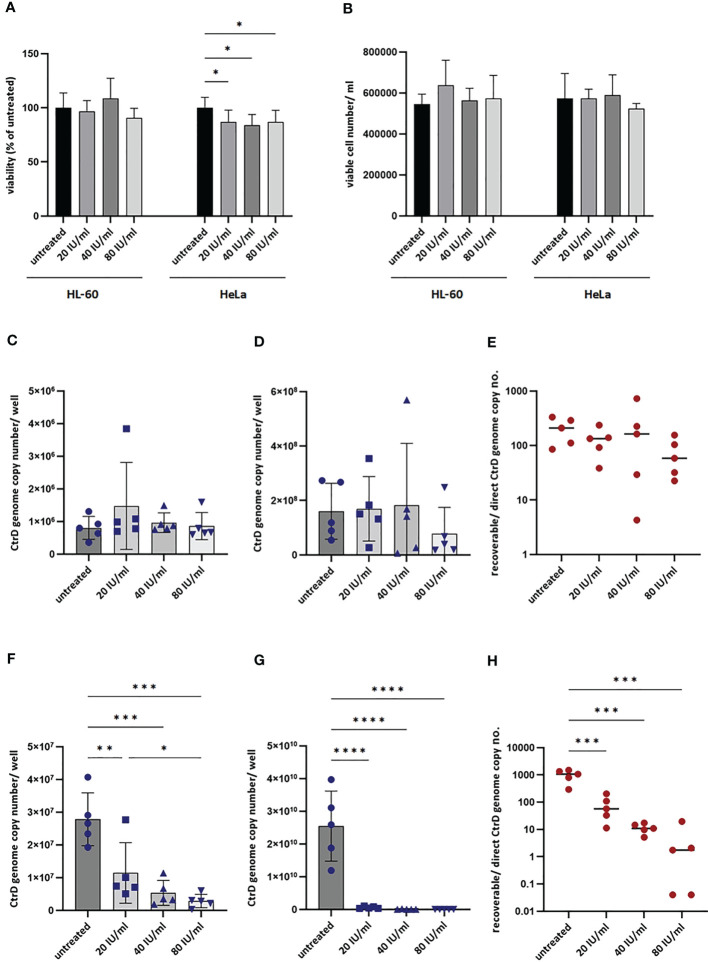
Impact of interferon-gamma (IFNG) on the growth of *Chlamydia trachomatis* in HL-60 and HeLa cells. **(A)** Impact of IFNG on the viability of HL-60 and HeLa cells. MTT cell viability assay of HL-60 and HeLa cells incubated with 0–20–40–80 IU/ml IFNG for 24 h. Data are mean ± SD (*n* = 8). **(B)** Viable cell counting of HL-60 and HeLa cells incubated with 0–20–40–80 IU/ml IFNG for 24 h. Data are mean ± SD (*n* = 4). To quantify *C. trachomatis* growth, HL-60 and HeLa cells were infected with *C. trachomatis* (multiplicity of infection, 4) and treated with 20–40–80 IU/ml IFNG. To measure direct chlamydial growth, HL-60 and HeLa cells (*n* = 5) were lysed at 48 h post-infection, and the chlamydial genome concentrations were measured by direct qPCR. For recoverable growth measurement, cell lysates from the direct growth measurements were used to infect McCoy cells. Chlamydial growth was measured by direct qPCR at 48 h post-infection. **(C)** Direct chlamydial growth in HL-60 cells. **(D)** Recoverable chlamydial growth in HL-60 cells. **(E)** Comparison of chlamydial genome concentration in recoverable *vs*. direct growth HL-60 samples. **(F)** Direct chlamydial growth in HeLa cells. **(G)** Recoverable chlamydial growth in HeLa cells. **(H)** Comparison of chlamydial genome concentration in recoverable *vs*. direct growth HeLa samples. For **(C, D, F, G)**, data are mean ± SD, and individual values are also shown (*n* = 5). For **(E, H)**, the mean and individual values are shown (*n* = 5). One-way ANOVA with correction for multiple testing was used for statistical analysis. *P < 0.033, **P < 0.002, ***P < 0.001, ****P < 0.0001.

**Figure 7:**
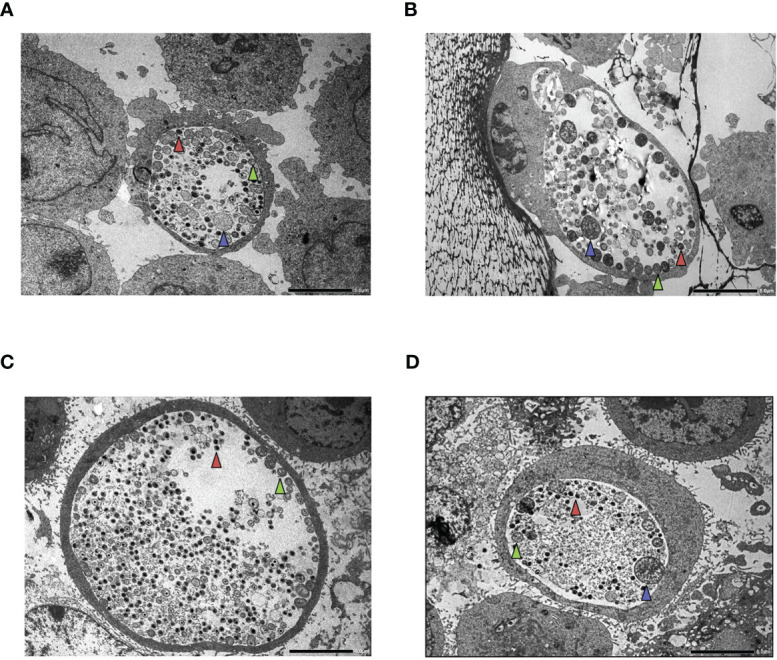
Transmission electron microscopy (TEM) of HL-60 and HeLa cells. Cells were infected with *Chlamydia trachomatis* (multiplicity of infection, 4) and were treated with 20 IU/ml interferon-gamma (IFNG) or left untreated. TEM was performed at 48 h post-infection. **(A)** *C. trachomatis*-infected HL-60 cell. **(B)** *C. trachomatis*-infected and IFNG-treated HL-60 cell. **(C)** *C. trachomatis*-infected HeLa cell. **(D)** *C. trachomatis*-infected and IFNG-treated HeLa cell. Red triangle, elementary body; green triangle, reticulate body; blue triangle, enlarged reticulate body or persistent body. Bar is 5 μm.

To exclude the antichlamydial effects of IFNG due to general cytotoxicity, we performed MTT based viability assays and trypan blue exclusion-based viable cell counts. These measurements showed that none of the IFNG concentrations had a significant impact on the viability of the HL-60 cells (**Figures 6A, B** **)**. The MTT viability assay of HeLa cells showed a moderate but concentration-independent decrease in cell reduction capacity by IFNG treatment (13.23–16.33% decrease compared to untreated control), but the number of viable cells did not change significantly by any of the applied IFNG concentrations (**Figures 6A, B** **)**. As an alternative of the immunofluorescence-based growth monitoring (23), we measured the chlamydial genome content at 48 h post-infection in both cell lines and also the recoverable chlamydial genome content in McCoy cells. Therefore, instead of direct IFU and recoverable IFU, we use the terms direct growth and recoverable growth. To test whether IFNG-induced IDO activity had an impact on chlamydial development, we treated HL-60 and HeLa cells with 0–20–40–80 IU/ml IFNG and measured the direct and recoverable chlamydial growth at 48 h post-infection. Independent of the applied concentration, IFNG had a non-significant impact on chlamydial genome accumulation in the infected HL-60 cells. The direct chlamydial growth was not affected by any of the IFNG concentrations, and we could not detect a significant decrease in recoverable chlamydial growth (**Figures 6C, D** **)**. Comparing the chlamydial genome content between the direct growth and recoverable growth samples, we could estimate a low-level (average 74.5–229.4-fold) accumulation of chlamydial genome in HL-60 cells (**Figure 6E**). The analysis of *C. trachomatis*-infected HeLa cells showed that these cells are more permissive for *C. trachomatis* growth; the chlamydial genome content was, on average, 34.56-fold higher in these cells than in untreated HL-60 cells (**Figure 6F**). The HeLa cells showed a dramatically different response to IFNG. Addition of IFNG significantly reduced the chlamydial genome contents (2.43–9.73-fold) (**Figure 6F**). The recoverable chlamydial growth was suppressed even more significantly. The extent of recoverable growth restriction was, on average, 44.34-fold (20 IU/ml IFNG), 388.4-fold (40 IU/ml IFNG), and 5,613-fold (80 IU/ml IFNG) (**Figure 6G**). Comparing the chlamydial genome content between the direct growth and recoverable growth samples revealed that the increase of chlamydial genome content was 1,000.31 ± 476-fold in the untreated HeLa cells. The increase of chlamydial genome content was reduced to averages of 82.37-, 11.52-, and 4.69-fold in the 20-, 40-, and 80-IU/ml-IFNG-treated samples, respectively (**Figure 6H**). We applied TEM to characterize chlamydial development in untreated and 20 IU/ml in treated HL-60 cells and as controls in HeLa cells. A comparison of the TEM images of *C. trachomatis*-infected and infected+IFNG-treated HL-60 cells showed similar chlamydial forms at 48 h post-infection. Intact inclusions could be observed with a small number of elementary bodies and reticulate bodies along with enlarged reticulate bodies or persistent bodies (**Figures 7A, B** **)**. The infected HeLa cells showed inclusions with predominantly elementary bodies in a large number, while the IFNG treatment induced enlarged chlamydial forms that resembled persistent bodies. However, a lower number of elementary bodies and reticulate bodies could also be observed (**Figures 7C, D** **)**.

## Discussion

In this study, we aimed to characterize the IDO-mediated neutrophil intracellular defense against *C. trachomatis* because, despite their short half-life, neutrophils can serve as host cells for *Chlamydia*. Zandbergen et al. showed that infection of human primary neutrophils by *C. pneumoniae* resulted in a moderate fivefold replication at 90 h post-infection, indicating that the intracellular environment may be permissive for chlamydial survival and a limited degree of growth (24). This is interesting because the neutrophils have potent antimicrobial mechanisms, such as reactive oxygen species (ROS) production and degranulation. Our gene expression profiling showed that many of these defense genes were upregulated after *C. trachomatis* infection, such as parts of the ROS-generating NADPH oxidase *cybb*, *ncf1*, and *ncf2* and various neutrophil granule-related catabolic enzymes such as *ctsh*, *galc*, *arsb*, *ids*, and *psap*. Besides these antimicrobial genes, the tryptophan-degrading enzyme coding genes *ido1* and the *tdo2* were found to be upregulated. It is widely accepted that IDO-mediated tryptophan degradation is an effective defense mechanism to inhibit chlamydial replication (8). IDO is an inducible enzyme, and while IDO induction by infection alone can be observed, IFNG is a potent inducer of its expression and activity (8). The gene expression profiling of HL-60 cells showed that *C. trachomatis* infection alone could induce type-I interferon expression and, *via* an autocrine–paracrine manner, a JAK-STAT cascade eventually leading to the induction of gene expression of several neutrophil genes. Indeed the promoter analysis of the induced genes showed a highly significant enrichment of interferon-related transcription factors. Among the infection-induced genes, *ido1* and *tdo2* were found, but for robust protein-level induction of IDO1, the addition of IFNG was needed. Our metabolomics data showed that *C. trachomatis* infection alone could induce a minor, non-significant IDO activity (increase of kynurenine concentration) in neutrophils, while the addition of exogenous IFNG greatly boosted the IDO activity. The HeLa cells showed a similar induction of IDO1 when compared to that in HL-60 cells, and a major factor affecting the IDO1 protein level expression was also the addition of exogenous IFNG. Despite the greatly induced IDO1 activity, the intracellular tryptophan levels changed only slightly compared to either the infected/untreated cells or the uninfected/untreated control cells in both cell lines. This observation is different from previous studies, where IFNG leads to a major decrease in intracellular tryptophan concentration (8, 25). The 20 IU/ml IFNG concentration used in our metabolic assays is equal to ≥1 ng/ml (26). Interestingly, Beatty et al. showed that the addition of 0.5 ng/ml IFNG leads to a less dramatic (53%) decrease in intracellular tryptophan in *C. trachomatis*-infected HeLa cells compared to infected/untreated controls (at 48 h post-infection) (7). Furthermore, they found that the intracellular tryptophan level decreased by 45% in *C. trachomatis*-infected + 0.5 ng/ml IFNG-treated HeLa cells compared to the infected-only cells. In our study, we measured a non-significant, but similar tryptophan concentration decrease (average 25%) in infected+IFNG-treated *vs*. infected-only HeLa cells. The intracellular tryptophan concentration is dependent on its net transport into the host cells and its usage in protein synthesis and catabolism. It was shown before that IFNG decreased the extracellular tryptophan concentration and greatly increased tryptophan transport into T24 human uroepithelial cells (27). In another study, the IFNG treatment of macaque macrophages resulted in a rapid drop in the extracellular tryptophan level, but the intracellular tryptophan concentration decreased moderately compared to the untreated ones, indicating that the missing tryptophan was replenished from the extracellular pool (28). An important limitation of these studies, including ours, is that only the total intracellular tryptophan concentration can be measured; thus, the tryptophan content of the inclusion is not known. In addition, there are differences between our study and the previous ones in sample preparation and tryptophan quantification method that might explain, to some extent, these results. Nevertheless, further studies are needed to clarify this difference.

The measurement of chlamydial genome accumulation in HL-60 and HeLa cells showed that, in the absence of IFNG, HeLa is highly permissive to chlamydial growth, while HL-60 supports a lower level but detectable replication. The chlamydial genome content was 34.56-fold higher in HeLa than in HL-60. Reticulate bodies and persistent bodies are not infectious; therefore, they do not contribute to recoverable growth. If we consider that the recoverable genome copy was 158.7 fold higher in samples of HeLa origin than in samples of HL-60 origin, we can conclude that there is a higher level of production of non-infectious or persistent chlamydial forms in HL-60 cells. The TEM images showed persistent chlamydial forms along with reticulate and elementary bodies in HL-60 cells, indicating at least a partially normal replication. Interestingly, chlamydial growth in HL-60 neutrophils was not influenced by the addition of IFNG despite IFNG-induced IDO activity in these cells. However, in HeLa cells, the addition of IFNG greatly inhibited the growth and the recoverable growth of *C. trachomatis*. The observed difference in tryptophan catabolism between HL-60 and HeLa cells might explain—at least in part—the differing inhibitory effect of IFNG. Narui et al. showed that the metabolites of tryptophan catabolism, such as 3-hydroxy-kynurenine, anthranilic acid, 3-hydroxyanthranilic acid, quinolinic acid, and especially picolinic acid, had an antimicrobial effect on Gram-positive and Gram-negative bacteria and *Candida albicans* (29). We showed that picolinic acid and 3-hydroxyanthranilic acid were produced in higher concentrations—2.36-fold and 13.37-fold, respectively—in infected+IFNG-treated HeLa cells than in infected+IFNG-treated HL-60 cells. Another potential antimicrobial compound, kynurenic acid (30), also had a 16.2-fold higher concentration in infected+IFNG-treated HeLa cells. Besides the different effect of antimicrobial tryptophan metabolites, there is also a possibility that HL-60 cells (31), but not HeLa cells, produced nitric oxide that could impair the function of IDO (32).

Altogether our data show that (i) the antichlamydial activity of IDO is cell type dependent and (ii) IFNG had a significant negative impact on *Chlamydia* growth in HeLa epithelial cells but had no antichlamydial activity in HL-60 neutrophils, indicating that neutrophils might serve as a refuge for *Chlamydia* in an IFNG-rich environment. Whether this cell-line based observation is valid in primary cells and *in vivo* needs more studies. Further studies on the intracellular context in which IDO functions and on tryptophan degradation-independent antichlamydial mechanisms are needed.

## Data Availability Statement

The gene expression raw data can be found here: Gene Expression Omnibus (Accession: GSE180238).

## Author Contributions

DV designed the experiments, analyzed the microarray data, and prepared the manuscript. SP performed the microarray experiments and low-level microarray data analysis. KB, VE, and AB were involved in designing the experiments. DK and DP were involved in *Chlamydia* propagation and manuscript preparation. AB and ZC performed tissue culturing and direct qPCR and were involved in manuscript preparation. FT, BB, and TJ performed the UHPLC–MS/MS measurements and data analysis. AK-P and KS performed Western-blot experiments, protein expression quantitation and were involved in the manuscript preparation. ZR performed the TEM analyses, evaluation of TEM images and was involved in the manuscript preparation. AK-P, KS and ZR approved the final revised form of the manuscript and agreed to be accountable for the accuracy and integrity of the data included in the manuscript. All authors contributed to the article and approved the submitted version.

## Funding

DV, TJ, FT, and BB were supported by EFOP 3.6.1 Program. TJ, FT, and BB were supported by the Thematic Excellence Program 2020 (TKP2020-IKA-07).

## Conflict of Interest

The authors declare that the research was conducted in the absence of any commercial or financial relationships that could be construed as a potential conflict of interest.

## Publisher’s Note

All claims expressed in this article are solely those of the authors and do not necessarily represent those of their affiliated organizations, or those of the publisher, the editors and the reviewers. Any product that may be evaluated in this article, or claim that may be made by its manufacturer, is not guaranteed or endorsed by the publisher.

## References

[1] Mohseni M Sung S Takov V. Chlamydia, in: StatPearls . Treasure Island (FL: StatPearls Publishing. Available at: http://www.ncbi.nlm.nih.gov/books/NBK537286/ (Accessed May 20, 2021).

[2] Morrison SG Morrison RP. *In Situ* Analysis of the Evolution of the Primary Immune Response in Murine Chlamydia Trachomatis Genital Tract Infection. Infect Immun (2000) 68:2870–9. doi: 10.1128/iai.68.5.2870-2879.2000 PMC97499 10768984

[3] Ficarra M Ibana JSA Poretta C Ma L Myers L Taylor SN. A Distinct Cellular Profile Is Seen in the Human Endocervix During Chlamydia Trachomatis Infection. Am J Reprod Immunol N Y N 1989 (2008) 60:415–25. doi: 10.1111/j.1600-0897.2008.00639.x PMC2574558 18798835

[4] Natividad A Freeman TC Jeffries D Burton MJ Mabey DCW Bailey RL. Human Conjunctival Transcriptome Analysis Reveals the Prominence of Innate Defense in Chlamydia Trachomatis Infection. Infect Immun (2010) 78:4895–911. doi: 10.1128/IAI.00844-10 PMC2976339 20823212

[5] Wong WF Chambers JP Gupta R Arulanandam BP. Chlamydia and Its Many Ways of Escaping the Host Immune System. J Pathog (2019) 2019:8604958. doi: 10.1155/2019/8604958 31467721 PMC6699355

[6] Lijek RS Helble JD Olive AJ Seiger KW Starnbach MN. Pathology After Chlamydia Trachomatis Infection Is Driven by Nonprotective Immune Cells That Are Distinct From Protective Populations. Proc Natl Acad Sci USA (2018) 115:2216–21. doi: 10.1073/pnas.1711356115 PMC5834673 29440378

[7] Beatty WL Belanger TA Desai AA Morrison RP Byrne GI. Tryptophan Depletion as a Mechanism of Gamma Interferon-Mediated Chlamydial Persistence. Infect Immun (1994) 62:3705–11. doi: 10.1128/IAI.62.9.3705-3711.1994 PMC303021 8063385

[8] Roshick C Wood H Caldwell HD McClarty G. Comparison of Gamma Interferon-Mediated Antichlamydial Defense Mechanisms in Human and Mouse Cells. Infect Immun (2006) 74:225–38. doi: 10.1128/IAI.74.1.225-238.2006 PMC1346650 16368976

[9] Virok DP Raffai T Kókai D Paróczai D Bogdanov A Veres G. Indoleamine 2,3-Dioxygenase Activity in Chlamydia Muridarum and Chlamydia Pneumoniae Infected Mouse Lung Tissues. Front Cell Infect Microbiol (2019) 9:192. doi: 10.3389/fcimb.2019.00192 31249813 PMC6582659

[10] Loomis WP Starnbach MN. T Cell Responses to Chlamydia Trachomatis. Curr Opin Microbiol (2002) 5:87–91. doi: 10.1016/s1369-5274(02)00291-6 11834375

[11] Tseng CT Rank RG. Role of NK Cells in Early Host Response to Chlamydial Genital Infection. Infect Immun (1998) 66:5867–75. doi: 10.1128/IAI.66.12.5867-5875.1998 PMC108743 9826367

[12] The Human Protein Atlas. Cell Type Atlas Ido1 . Available at: https://www.proteinatlas.org/ENSG00000131203-IDO1/celltype.

[13] Caldwell HD Kromhout J Schachter J. Purification and Partial Characterization of the Major Outer Membrane Protein of Chlamydia Trachomatis. Infect Immun (1981) 31:1161–76. doi: 10.1128/IAI.31.3.1161-1176.1981 PMC351439 7228399

[14] Millius A Weiner OD. Manipulation of Neutrophil-Like HL-60 Cells for the Study of Directed Cell Migration. Methods Mol Biol Clifton NJ (2010) 591:147–58. doi: 10.1007/978-1-60761-404-3_9 PMC3128798 19957129

[15] Sherman BT Huang DW Tan Q Guo Y Bour S Liu D. DAVID Knowledgebase: A Gene-Centered Database Integrating Heterogeneous Gene Annotation Resources to Facilitate High-Throughput Gene Functional Analysis. BMC Bioinf (2007) 8:426. doi: 10.1186/1471-2105-8-426 PMC2186358 17980028

[16] Raudvere U Kolberg L Kuzmin I Arak T Adler P Peterson H. G:Profiler: A Web Server for Functional Enrichment Analysis and Conversions of Gene Lists (2019 Update). Nucleic Acids Res (2019) 47:W191–8. doi: 10.1093/nar/gkz369 PMC6602461 31066453

[17] Tömösi F Kecskeméti G Cseh EK Szabó E Rajda C Kormány R. A Validated UHPLC-MS Method for Tryptophan Metabolites: Application in the Diagnosis of Multiple Sclerosis. J Pharm BioMed Anal (2020) 185:113246. doi: 10.1016/j.jpba.2020.113246 32182446

[18] Tuka B Nyári A Cseh EK Körtési T Veréb D Tömösi F. Clinical Relevance of Depressed Kynurenine Pathway in Episodic Migraine Patients: Potential Prognostic Markers in the Peripheral Plasma During the Interictal Period. J Headache Pain (2021) 22:60. doi: 10.1186/s10194-021-01239-1 34171996 PMC8229298

[19] Mosmann T. Rapid Colorimetric Assay for Cellular Growth and Survival: Application to Proliferation and Cytotoxicity Assays. J Immunol Methods (1983) 65:55–63. doi: 10.1016/0022-1759(83)90303-4 6606682

[20] Eszik I Lantos I Önder K Somogyvári F Burián K Endrész V. High Dynamic Range Detection of Chlamydia Trachomatis Growth by Direct Quantitative PCR of the Infected Cells. J Microbiol Methods (2016) 120:15–22. doi: 10.1016/j.mimet.2015.11.010 26578244

[21] Dhanasekaran S Doherty TM Kenneth J. TB Trials Study Group. Comparison of Different Standards for Real-Time PCR-Based Absolute Quantification. J Immunol Methods (2010) 354:34–9. doi: 10.1016/j.jim.2010.01.004 20109462

[22] Ibana JA Belland RJ Zea AH Schust DJ Nagamatsu T AbdelRahman YM. Inhibition of Indoleamine 2,3-Dioxygenase Activity by Levo-1-Methyl Tryptophan Blocks Gamma Interferon-Induced Chlamydia Trachomatis Persistence in Human Epithelial Cells. Infect Immun (2011) 79:4425–37. doi: 10.1128/IAI.05659-11 PMC3257928 21911470

[23] Ouellette SP Hatch TP AbdelRahman YM Rose LA Belland RJ Byrne GI. Global Transcriptional Upregulation in the Absence of Increased Translation in Chlamydia During IFNgamma-Mediated Host Cell Tryptophan Starvation. Mol Microbiol (2006) 62:1387–401. doi: 10.1111/j.1365-2958.2006.05465.x 17059564

[24] van Zandbergen G Gieffers J Kothe H Rupp J Bollinger A Aga E. Chlamydia Pneumoniae Multiply in Neutrophil Granulocytes and Delay Their Spontaneous Apoptosis. J Immunol Baltim Md 1950 (2004) 172:1768–76. doi: 10.4049/jimmunol.172.3.1768 14734760

[25] Kane CD Vena RM Ouellette SP Byrne GI. Intracellular Tryptophan Pool Sizes may Account for Differences in Gamma Interferon-Mediated Inhibition and Persistence of Chlamydial Growth in Polarized and Nonpolarized Cells. Infect Immun (1999) 67:1666–71. doi: 10.1128/IAI.67.4.1666-1671.1999 PMC96511 10085001

[26] Peprotech Recombinant Human IFNG Data Sheet. Available at: https://www.peprotech.com/en/recombinant-human-ifn-2-2.

[27] Byrne GI Lehmann LK Landry GJ. Induction of Tryptophan Catabolism Is the Mechanism for Gamma-Interferon-Mediated Inhibition of Intracellular Chlamydia Psittaci Replication in T24 Cells. Infect Immun (1986) 53:347–51. doi: 10.1128/iai.53.2.347-351.1986 PMC260881 3089936

[28] Drewes JL Croteau JD Shirk EN Engle EL Zink MC Graham DR. Distinct Patterns of Tryptophan Maintenance in Tissues During Kynurenine Pathway Activation in Simian Immunodeficiency Virus-Infected Macaques. Front Immunol (2016) 7:605. doi: 10.3389/fimmu.2016.00605 28066416 PMC5165277

[29] Narui K Noguchi N Saito A Kakimi K Motomura N Kubo K. Anti-Infectious Activity of Tryptophan Metabolites in the L-Tryptophan-L-Kynurenine Pathway. Biol Pharm Bull (2009) 32:41–4. doi: 10.1248/bpb.32.41 19122278

[30] Dolecka J Urbanik-Sypniewska T Skrzydło-Radomańska B Parada-Turska J. Effect of Kynurenic Acid on the Viability of Probiotics *In Vitro* . Pharmacol Rep PR (2011) 63:548–51. doi: 10.1016/s1734-1140(11)70522-9 21602611

[31] Kawase T Orikasa M Oguro A Burns DM. Up-Regulation of Inducible Nitric Oxide (NO) Synthase and NO Production in HL-60 Cells Stimulated to Differentiate by Phorbol 12-Myristate 13-Acetate Plus 1,25-Dihydroxyvitamin D3 Is Not Obtained With Dimethylsulfoxide Plus 1,25-Dihydroxyvitamin D3. Calcif Tissue Int (1998) 63:27–35. doi: 10.1007/s002239900485 9632843

[32] Thomas SR Mohr D Stocker R. Nitric Oxide Inhibits Indoleamine 2,3-Dioxygenase Activity in Interferon-Gamma Primed Mononuclear Phagocytes. J Biol Chem (1994) 269:14457–64. doi: 10.1016/S0021-9258(17)36645-0 7514170

