# Supplementary material for: Indoleamine 2,3-Dioxygenase Cannot Inhibit Chlamydia trachomatis Growth in HL-60 Human Neutrophil Granulocytes
Source: Front Immunol. 2021 Nov 8;12:717311. doi: 10.3389/fimmu.2021.717311 (PMC8606673; doi:10.3389/fimmu.2021.717311)
Supplement: Supplementary file 1 [file DataSheet_1.docx]

**UHPLC-MS/MS reagents and chemicals.** All reagents and chemicals were analytical or LC–MS grade. L-tryptophan and its metabolites, d4-picolinic acid, and n-butanol were purchased from Sigma-Aldrich (St. Louis, MO, USA). d3-3-hydroxykynurenine was obtained from Buchem B. V. (Apeldoorn, The Netherlands). The other deuterated internal standards (IS; d4-serotonin, d4-L-kynurenine, d3-3-hydroxyanthranilic acid, d5-tryptophan, d5-5-hydroxyindoleacetic acid, d5-kynurenic acid, d4-xanthurenic acid, d4-melatonin, and d3-quinolinic acid) were purchased from Toronto Research Chemicals (Toronto, ON, Canada). Acetonitrile (ACN), methanol (MeOH), and water were obtained from VWR Chemicals (Monroeville, PA, USA). Formic acid (FA) and hydrochloric acid was purchased from Fisher Scientific (Portsmouth, NH, USA), and acetyl chloride was obtained from Alfa Aesar (Haverhill, MA, USA).

**Preparation of standard, internal standard (IS), and quality control (QC) solutions.** The preparation of the stock solutions of L-tryptophan (TRP) and its metabolites was detailed in our previous publication (Tömösi et al., 2020). All standard stock solutions were prepared on ice, divided into 100-µL aliquots, and stored at −80°C until further use. Calibration standards for the tryptophan and its 11 metabolites analysis were prepared at 12 levels, and the QC samples were prepared at three levels (low level QC [LQC]; middle level QC [MQC]; and high level QC [HQC], Supplementary Table 1) in PBS. Calibration standards consisted of 90 μL of PBS, 10 μL of standard solution mix (serotonin (SERO), L-kynurenine (KYN), 3-hydroxyanthranilic acid (3-HANA), TRP, 5-hydroxyindoleacetic acid (5-HIAA), kynurenic acid (KYNA), 3-hydroxykynurenine (3-HK), xanthurenic acid (XA), melatonin (MELA), picolinic acid (PICA), and quinolinic acid (QUIN)) in 0.1% (v/v) aqueous formic acid (FA), and 300 μL of ice-cold acetonitrile (ACN) containing 10 μL of the stable isotope-labeled (SIL)-IS mix (10 ng/mL d4-SERO, 10 ng/mL d4-KYN, 1 ng/mL d3-3-HANA, 50 ng/mL d5-TRP, 10 ng/mL d5-5-HIAA, 1 ng/mL d5-KYNA, 2 ng/mL d3-3-HK, 1 ng/mL d4-XA, 1 ng/mL d4-MELA, 2.5 ng/mL d4-PICA, and 8 ng/mL d3-QUIN).

**UHPLC-MS/MS method validation, linearity, limit of detection (LOD), limit of quantification (LOQ).** In this study, SIL-IS analogs were used to validate the UHPLC–MS/MS method. For the validation process, the linearity, LOD, LOQ, precision and accuracy were assessed following the official guidelines (Borman and Elder, 2017) (Bioanalytical Method Validation Guidance for Industry). The calibration curves of the analytes were constructed from the peak area ratios of the compound to SIL-IS at 12 levels using mixed working standard solutions. According to the acceptance criteria, the calibration curve should have a correlation coefficient (r2) of 0.99 or better. The LOD and LOQ were calculated using the formulae 3.3∙α/S and 10∙α/S, respectively, where α is the standard error of the y-intercept and S is the slope of the calibration curve (Supplementary Table 2). The UHPLC-MS/MS accuracy and intra-day precisions were calculated by determining four replicates of LQC, MQC, and HQC samples (n = 12). For precision, the acceptance criterion was ±15% with respect to the relative standard deviation (RSD). For accuracy, the limit was the same relative to the definite concentrations. The RSD and accuracy values are presented in Supplementary Table 1.

**References**

Bioanalytical Method Validation Guidance for Industry Available at: https://www.fda.gov/files/drugs/published/Bioanalytical-Method-Validation-Guidance-for-Industry.pdf.

Borman, P., and Elder, D. (2017). “Q2(R1) Validation of Analytical Procedures,” in *ICH Quality Guidelines* (John Wiley & Sons, Ltd), 127–166. doi:10.1002/9781118971147.ch5.

Tömösi, F., Kecskeméti, G., Cseh, E. K., Szabó, E., Rajda, C., Kormány, R., et al. (2020). A validated UHPLC-MS method for tryptophan metabolites: Application in the diagnosis of multiple sclerosis. *J. Pharm. Biomed. Anal.* 185, 113246. doi:10.1016/j.jpba.2020.113246.
