# Supplementary material for: Indoleamine 2,3-Dioxygenase Cannot Inhibit Chlamydia trachomatis Growth in HL-60 Human Neutrophil Granulocytes
Source: Front Immunol. 2021 Nov 8;12:717311. doi: 10.3389/fimmu.2021.717311 (PMC8606673; doi:10.3389/fimmu.2021.717311)
Supplement: Supplementary file 2 [file Table_1.docx]

Supplementary Table 1. Concentrations, intra-day precision and accuracy of TRP and its metabolites at low, medium, and high levels.

|  | LQC | | | MQC | | | HQC | | |
| --- | --- | --- | --- | --- | --- | --- | --- | --- | --- |
| Analytes | Concentration (ng/ml) | RSD% | Accuracy% | Concentration (ng/ml) | RSD% | Accuracy% | Concentration (ng/ml) | RSD% | Accuracy% |
| SERO | 0.70 | 8.5 | 100.4 | 3.75 | 2.9 | 97.7 | 7.50 | 3.6 | 98.7 |
| KYN | 3.52 | 5.0 | 97.2 | 18.75 | 2.5 | 99.6 | 37.50 | 2.1 | 101.9 |
| 3-HANA | 0.18 | 9.5 | 96.1 | 0.94 | 3.4 | 94.4 | 1.88 | 2.5 | 104.0 |
| TRP | 7.03 | 7.9 | 102.5 | 37.50 | 5.5 | 104.0 | 75.00 | 3.7 | 102.0 |
| 5-HIAA | 0.70 | 3.1 | 94.0 | 3.75 | 3.3 | 95.0 | 7.50 | 2.5 | 95.6 |
| ANA | 0.70 | 16.8 | 92.5 | 3.75 | 5.3 | 85.7 | 7.50 | 5.7 | 101.6 |
| KYNA | 0.07 | 6.9 | 93.0 | 0.38 | 2.9 | 92.1 | 0.75 | 5.8 | 86.4 |
| XA | 0.07 | 6.0 | 95.6 | 0.38 | 3.9 | 101.6 | 0.75 | 1.5 | 100.3 |
| 3-HK | 0.14 | 14.1 | 103.5 | 0.75 | 4.7 | 111.1 | 1.50 | 8.7 | 95.8 |
| MELA | 0.70 | 3.5 | 101.5 | 3.75 | 2.1 | 98.9 | 7.50 | 3.5 | 100.3 |
| PICA | 0.18 | 4.2 | 93.3 | 0.94 | 5.4 | 95.1 | 1.88 | 2.3 | 95.5 |
| QUIN | 1.76 | 0.6 | 106.8 | 9.38 | 3.0 | 97.7 | 18.75 | 2.1 | 99.5 |

RSD, relative standard deviation; LQC, low-level quality control; MQC, medium-level quality control; HQC, high-level quality control; SERO, serotonin; KYN, l-kynurenine; 3-HANA, 3-hydroxyanthranilic acid; TRP, tryptophan; 5-HIAA, 5-hydroxyindoleacetic acid; ANA, anthranilic acid; KYNA, kynurenic acid; XA, xanthurenic acid; 3-HK, 3-hydroxykynurenine; MELA, melatonin; PICA, picolinic acid; QUIN, quinolinic acid.
