# Supplementary material for: Indoleamine 2,3-Dioxygenase Cannot Inhibit Chlamydia trachomatis Growth in HL-60 Human Neutrophil Granulocytes
Source: Front Immunol. 2021 Nov 8;12:717311. doi: 10.3389/fimmu.2021.717311 (PMC8606673; doi:10.3389/fimmu.2021.717311)
Supplement: Supplementary file 3 [file Table_2.docx]

Supplementary Table 2. LODs, LOQs and the parameters of the calibration curves for TRP and its metabolites.

| Analytes | LOD | LOQ | Regression correlation | Linearity range |
| --- | --- | --- | --- | --- |
|  | ng/ml | |  | ng/ml |
| SERO | 0.082 | 0.249 | 0.9992 | 0.31-10 |
| KYN | 0.087 | 0.264 | 0.9995 | 1.56-50 |
| 3-HANA | 0.014 | 0.042 | 0.9996 | 0.07-2.5 |
| TRP | 0.568 | 1.720 | 0.9992 | 3.12-100 |
| 5-HIAA | 0.056 | 0.168 | 0.9996 | 0.31-10 |
| ANA | 0.056 | 0.169 | 0.9986 | 0.30-10 |
| KYNA | 0.006 | 0.018 | 0.9996 | 0.03-1.0 |
| XA | 0.005 | 0.015 | 0.9990 | 0.03-1.0 |
| 3-HK | 0.048 | 0.145 | 0.9979 | 0.25-2.0 |
| MELA | 0.045 | 0.135 | 0.9998 | 0.31-10.0 |
| PICA | 0.028 | 0.086 | 0.9996 | 0.07-2.5 |
| QUIN | 0.143 | 0.432 | 0.9998 | 1.56-25.0 |

LOD, limit of detection; LOQ, limit of quantification; SERO, serotonin; KYN, l-kynurenine; 3-HANA, 3-hydroxyanthranilic acid; l-TRP, tryptophan; 5-HIAA, 5-hydroxyindoleacetic acid; ANA, anthranilic acid; KYNA, kynurenic acid; XA, xanthurenic acid; 3-HK, 3-
